# A Man in His Forties with Recurrent Cat-Scratch Disease

**DOI:** 10.1155/2024/4411133

**Published:** 2024-02-27

**Authors:** Sara López-Rueda, Benjamin Valente-Acosta, Adrian Murillo-Zolezzi, Francisco Moreno-Sánchez, Irma Hoyo-Ulloa, Jesús Javier Baquera-Heredia

**Affiliations:** The American British Cowdray Medical Center, Sur 136 116, Alvaro Obregon, Mexico City 01120, Mexico

## Abstract

Cat-scratch disease (CSD) is a self-limited zoonotic infection transmitted by felines caused by the Gram-negative bacillus *Bartonella henselae*. It usually presents with lymphadenopathy and constitutional symptoms that resolve within eight weeks, with, or without antibiotic treatment. The diagnosis is made by serology, molecular diagnosis in a biopsy, or a positive culture. The recurrence or reactivation of *B. henselae* has rarely been reported. We present the case of a 45-year-old man with a history of CSD two years before who presented to the clinic with groin lymphadenopathy. The patient had a history of close contact with felines though no known risk exposure was reported. The diagnosis was made with a positive serology suggestive of recent infection along with histopathological changes suggestive of CSD. Subsequently, azithromycin was administered with complete resolution of symptoms.

## 1. Background

Cat-scratch disease (CSD) is a zoonotic infection caused by the Gram-negative bacillus *Bartonella henselae*. The infection is transmitted by cats, especially those younger than one year old, through bites and scratches [1, 2]. It is usually a benign and self-limited disease that presents with regional unilateral lymphadenopathy proximal to the injured site, constitutional symptoms, and a history of previous intimate contact with a cat [3]. Atypical CSD can present with signs and symptoms of hepatic, ocular, and central nervous system involvement, as well as other severe and possibly life-threatening manifestations such as fever of unknown origin [4]. CSD can be diagnosed by clinical examination, serologic, molecular methods, and tissue biopsy. CSD is usually treated with antibiotics, such as azithromycin, to reduce lymphadenopathy and symptom duration.

## 2. Case Presentation

A 45-year-old Hispanic man with a history of CSD that was diagnosed two years previously, presented to the outpatient consult with dyspnoea, diaphoresis, fatigue, pharyngodynia, and bilateral inguinal lymph nodes' enlargement for the past three months. His past medical history is relevant for mononucleosis when he was 24 years old. He denied unsafe sexual practices and travelling abroad. He frequently visited wooded areas for hiking.

The patient owned three dogs and had a history of contact with cats, tigers, lions, and farm animals. However, the patient did not recall receiving recent bites or scratches from cats or dogs other than a feral kitten bite that occurred two years ago in his left hand when he was first diagnosed with CSD.

Two years ago, he presented with a swollen hand with subsequent axillar lymph nodes' enlargement. The diagnosis was based on a left axillar lymph node biopsy made in another hospital. It showed changes related to *B. henselae* infection, although neither serological nor molecular investigations were conducted at that time. He was treated successfully with an antibiotic, but the patient did not recall its name, and his medical records from the other clinic were not available.

**Figure 1:**
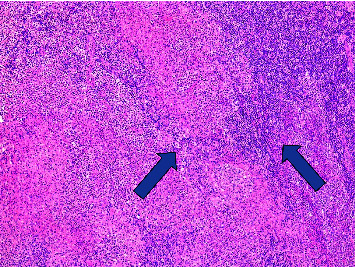
Lymph node showing granulomatous and suppurative lymphadenitis, low power view (granulomatous component marked with arrow).

**Figure 2:**
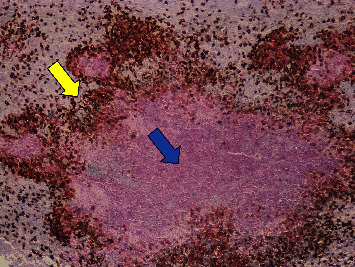
Double immunohistochemical staining for CD163 (brown chromogen, yellow arrow) highlighted the granulomatous component, and MPO (red chromogen, blue arrow) highlighted the suppurative part of the tissue response (H & E, 100x).

Two months before our initial evaluation, the patient had consulted an oncologist because of malaise, fatigue, odynophagia, and axillary pruritus, followed by enlarged and tender inguinal lymph nodes. He underwent a right inguinal lymphadenectomy a month later, with histopathologic findings of acute suppurative lymphadenitis with granulomatous components suggestive of bacterial infection (Figure 1), but no specific microorganisms were identified by a Warthin–Starry stain (Figure 2).

The biopsy results were negative for TB staining and molecular detection. A week after the right inguinal lymphadenectomy, the patient presented with lymphorrhagia, which was successfully treated with one dose of intramuscular long-lasting octreotide (20 mg) and wound pressure.

At the initial physical examination, the patient had bilateral inguinal lymphadenopathy, scrotal swelling, and conjunctival hyperaemia; no hepatosplenomegaly was noted. His right groin had a scar with a drain that had persistent lymph discharge. At the time of the initial evaluation, the patient had been taking doxycycline for five days prescribed by the surgeon but persisted with constitutional symptoms, diaphoresis, dyspnoea, and pharyngodynia.

The results of the initial laboratory work-up were within normal ranges, including complete blood count, electrolytes, C-reactive protein (0.16 mg/dL, reference value 0.00–0.50), and erythrocyte sedimentation rate (11 mm/hr, reference value 0–20). The comprehensive metabolic panel showed a mild increase in alanine aminotransferase, and the rest of the liver enzymes were normal. p-ANCA and c-ANCA were negative. On the chest X-ray, there was a small 4-mm calcified granuloma at the right lower pulmonary lobe; otherwise, there were no abnormal findings.

Serologies for VDRL, HIV, Epstein–Barr, *Toxoplasma*, and *Brucella* spp were nonreactive. *B. henselae* IgG was suggestive of a recent infection (1 : 512, negative <1 : 20), *B. henselae* IgM was negative (<1 : 20), *B. quintana* IgG was slightly abnormal (1 : 128, negative <1 : 20), and IgM was negative. *B. henselae* polymerase chain reaction (PCR) was negative in the blood and in the groin lymphadenectomy specimen.

After the initial assessment, a prescription of azithromycin 500 mg once daily for five days was given. During the follow-up visit after one month, the symptoms had resolved. The physical examination revealed reduced inguinal lymphadenopathy and decreased scrotal swelling. It was assumed that the patient had positively responded to the antimicrobial treatment with azithromycin, as evidenced by the resolution of symptoms and cessation of lymphorrhagia.

## 3. Discussion

CSD is the most common clinical presentation of *Bartonella henselae* infections. The infection is most commonly transmitted to humans by cats. The risk of transmission between humans and cats has not been determined. However, a study conducted in Turkey found an 11.5% seroprevalence in a simultaneously assessed sample in cat/dog [5]. Additionally, in Chile, 18.1% of owners with positive cats (71.0%) exhibited antibodies against *B. henselae* [6].

It is generally a self-limited disease; 80% to 90% of cases present with regional unilateral lymphadenopathy proximal to the inoculation site and flu-like symptoms that resolve within two to eight weeks [3]. Studies have shown that 90% of patients had resolution of lymphadenopathy within 16 weeks [7]. Although the disease usually has a benign course, there can be hematogenous spread to other organs, causing a wide spectrum of atypical presentations that can occur in 10–15% of cases [8]. Immunocompromised patients are at a greater risk for severe systemic CSD [9].

The recurrence of cat-scratch disease has rarely been reported. To the best of our knowledge, there are few case reports and a case series of CSD lymphadenitis recurrence in previously healthy patients [10, 11]. CSD relapse in immunocompromised patients has been reported in a kidney recipient patient [12]. Ocular recurrences have been reported [13, 14], as well as persistent lymphadenopathy and lymphadenitis [7, 14].

In 1965, Townsend and Cravitz described the first case of CSD recurrence in a 13-year-old female who was previously healthy. The patient presented with suppurative inguinal lymphadenitis two years after the initial diagnosis and had no history of cat scratches or bites. The authors concluded that it probably resulted from a flare-up of the prior infection, but they did not rule out the possibility of reinfection [11].

In 1987, Margileth et al. reported a series of 23 cases of prolonged or recurrent CSD and confirmed two cases of recurrent CSD based on finding *B. henselae* in lymph node biopsy specimens. They reported recurrence at 4- to 20-month intervals. They also described an apparent recurrence in an adult patient who presented with inguinal lymphadenitis 10 months after the initial diagnosis. The lymph nodes were Warthin–Starry negative like in our case, and the patient had a one-month history of fever, malaise, fatigue, headache, and weight loss [10].

Immunocompromised patients are at a greater risk of severe and prolonged disease. Rheault et al. reported a CSD relapse in a 19-year-old male kidney transplant recipient [12]. Persistent CSD has also been described in immunosuppressed patients and as causing recurrent fever in patients with HIV infection [3]. Our patient had no laboratory or clinical evidence of immunosuppression; he was nonreactive for HIV, and routine blood tests were within normal ranges.

The origin of our patient's second insult could have been a flare-up from the previous infection or reinfection, although he denied having had a cat scratch or bite. It is well known that CSD can occur without a history of cat exposure because the cat flea (*Ctenocephalides felis*), as a vector, can transmit the infection through its faeces [15]. Twenty-five per cent of patients diagnosed with CSD have no intimate contact with cats [8]. The possibility that our patient was reinfected cannot be ruled out due to the repeated contact with felines in the two years after the first CSD. However, the serology is suggestive of recurrence or relapse due to the high IgG titters and negative IgM.

Serology is the first-line diagnostic test for CSD; commercial Indirect Fluorescent Antibody Essays (IFAEs) can detect *B. henselae* IgM and IgG antibodies [16]. Antibody titters peak at four to five months and can persist for up to three years [17]. IgG titters greater than 1 : 256 are suggestive of an active or recent infection [3]. Detection of *B. henselae* DNA in lymph nodes and blood by PCR is also widely used for the laboratory diagnosis of CSD, and it is especially useful in seronegative patients [18], although the sensitivity is not 100%.

Regrettably, Bartonella's serology or PCR was not conducted in the first episode; the diagnosis was based on histopathological images within a history exposure to a cat. In a recent presentation, *B. henselae* IgG titters were suggestive of recent or active infection (1 : 512), PCR was negative, and *B. henselae* was not identified in lymph node biopsy, similar to the case reported by Margileth et al. [10].

Although lymph node biopsy is important, it is not a routine procedure in classic self-limited cases. The characteristic histopathological findings are stellate microabscesses surrounded by granulomatous inflammation [19]. On silver impregnation stains, such as Warthin–Starry, a pleomorphic rod can be seen in the early stages of the disease [8, 19].

There are no guidelines or consensus regarding the treatment of *B. henselae*, which is based mainly on case reports. Treatment varies among the various clinical manifestations of CSD; however, no single treatment works against all types of CSD-associated diseases [20]. In self-limited cases, antibiotics are not usually given, and the treatment is based on analgesia and symptom resolution. In patients with lymphadenopathy that does not resolve over the expected period of 16 weeks and who have persistent and limiting symptoms, azithromycin can be used for two to five days [9]. Azithromycin has been shown to be effective in decreasing lymph node size in the first month and is considered the treatment of choice [21]. Doxycycline combined with rifampin is an alternative [22]. Lymph nodes should be drained or aspirated in cases of lymphadenitis to help with the differential diagnosis [9]. Lymph node aspiration should be reserved for severe cases to rule out other possible and more serious diagnoses [8].

To the best of our knowledge, no previous study has shown the effectiveness of prolonged antimicrobial treatment in preventing recurrence, reactivation, or reinfection. Further studies are needed to determine the efficacy of prolonged treatment to prevent cases such as those reported here. Prolonged *B. henselae* lymphadenitis that did not resolve with prolonged antimicrobial treatment has been reported [7]. This was different from our case because the patient responded to the first antimicrobial treatment, remained asymptomatic, and had no lymphadenitis or lymphadenopathy in the two-year period between episodes.

Our case is relevant because it describes an uncommon recurrence of CSD. The recurrence of CSD is rare and had been seldom reported. Also, the report underscores the importance of considering CSD in patients with similar symptoms, even if they have a history of successful treatment for a previous infection.

## 4. Conclusions

CSD usually has a benign course and resolves without further complications, but it is possible for the infection to recur or reactivate even in absence of intimate contact with cats. In patients with a history of CSD with lymphadenopathy and lymphadenitis reinfection, recurrence or reactivation of *B. henselae* should be considered as a possible diagnosis and lymph node aspiration or draining should be made to rule out other serious diagnosis since CSD can mimic several severe and malignant conditions.

## Data Availability

All data generated or analyzed during this study are included in this article and are available upon reasonable request to the corresponding author.

## References

[1] Chomel B. B., Boulouis H. J., Petersen H. (2002). Prevalence of Bartonella infection in domestic cats in Denmark. *Veterinary Research*.

[2] Fleischman D. A., Chomel B. B., Kasten R. W. (2015). Bartonella infection among cats adopted from a San Francisco shelter, revisited. *Applied and Environmental Microbiology*.

[3] Klotz S. A., Ianas V., Elliott S. P. (2011). Cat-scratch disease. *American Family Physician*.

[4] Landes M., Maor Y., Mercer D. (2020). Cat scratch disease presenting as fever of unknown origin is a unique clinical syndrome. *Clinical Infectious Diseases*.

[5] Aydın N., Korkmazgil B., Kırkan Ş. (2019). Seropositivity of bartonella henselae in risky human population, cats and dogs. *Meandros Medical and Dental Journal*.

[6] Zaror L., Ernst S., Navarrete M. (2002). Detección serológica de Bartonella henselae en gatos en la ciudad de Valdivia, Chile. *Archivos de Medicina Veterinaria*.

[7] King K. Y., Hicks M. J., Mazziotti M. V., Eldin K. W., Starke J. R., Michael M. (2015). Persistent cat scratch disease requiring surgical excision in a patient with MPGN. *Pediatrics*.

[8] Zangwill K. M. (2021). Cat scratch disease and bartonellaceae: the known, the unknown and the curious. *The Pediatric Infectious Disease Journal*.

[9] Blagova B., Yanev N. (2021). Human bartonella infection: a review of literature. *Journal of Annual Assembly of International Medical Association Bulgaria- Annual Proceeding (Scientific Papers)*.

[10] Margileth A. M., Wear D. J., English C. K. (1987). Systemic cat scratch disease: report of 23 patients with prolonged or recurrent severe bacterial infection. *Journal of Infectious Diseases*.

[11] Townsend E. H., Cravitz L. (1965). Cat-scratch disease recurrence after three years. *American Journal of Diseases of Children*.

[12] Rheault M. N., Van Burik J. A., Mauer M. (2007). Cat-scratch disease relapse in a kidney transplant recipient. *Pediatric Transplantation*.

[13] Sundaram S. V., Purvin V. A., Kawasaki A. (2012). The clinical profile of idiopathic and cat scratch neuroretinitis: who is at risk for recurrence. *Neuro-Ophthalmology*.

[14] Ng C. C., Ng J., McDonald H. R., Cunningham E. T. (2022). Bartonella henselae-associated recurrent, bilateral segmental periphlebitis. *American Journal of Ophthalmology Case Reports*.

[15] Oka K., Takagi Y., Hagiya H., Otsuka F. (2022). Cat scratch disease without a history of cat exposure. *Clinical Case Reports*.

[16] Allizond V., Costa C., Sidoti F. (2019). Serological and molecular detection of Bartonella henselae in specimens from patients with suspected cat scratch disease in Italy: a comparative study. *PLoS One*.

[17] Zangwill K. M. (2013). Cat scratch disease and other bartonella infections. *Advances in Experimental Medicine and Biology*.

[18] Yanagihara M., Tsuneoka H., Tanimoto A. (2018). Bartonella henselae DNA in seronegative patients with cat-scratch disease. *Emerging Infectious Diseases*.

[19] Lamps L. W., Scott M. A. (2004). Cat-scratch disease. *Pathology Patterns Reviews*.

[20] Kumar S., Gadila G., Embers M. E., Breitschwerdt B. (2021). Antibiotic susceptibility of bartonella grown in different culture conditions. *Pathogens*.

[21] Biswas S., Rolain J. M. (2010). Bartonella infection: treatment and drug resistance. *Future Microbiology*.

[22] Rolain J. M., Brouqui P., Koehler J. E., Maguina C., Dolan M. J., Raoult D. (2004). Recommendations for treatment of human infections caused by Bartonella species. *Antimicrobial Agents and Chemotherapy*.

